# Sternal metastasis as an initial presentation of renal cell carcinoma: a case report

**DOI:** 10.4076/1757-1626-2-9045

**Published:** 2009-09-10

**Authors:** Raquel Ribeiro Batista, Edson Marchiori, Tatiana Chinem Takayassu, Fernanda Caseira Cabral, Rafael Ferracini Cabral, Gláucia Zanetti

**Affiliations:** 1 Service of Diagnostic Radiology, Clementino Fraga Filho Universitary HospitalRua Prof. Rodolpho Paulo Rocco, 225. CEP 21.941.913. Cidade Universitária. Rio de JaneiroBrazil; 2 Department of Radiology, Faculty of Medicine, Fluminense Federal UniversityRua Marquês do Paraná, 303. CEP 24.033.900. Niterói. Rio de JaneiroBrazil

## Abstract

Renal cell carcinoma accounts for 85% of all solid renal tumors in adults. Nearly one quarter of patients has distant metastasis at presentation while another 50% develop metastasis during follow-up. A small percentage of these are solitary metastasis. We report here a case of solitary bone sternal metastasis as an initial presentation of clear-cell renal cell carcinoma in a 56-year-old woman. The prognosis for patients with metastasized renal cell carcinoma is poor; treatment of metastasis is usually palliative and designed to provide comfort and pain relief. Palliative nephrectomy may be considered for control of symptoms. Radical nephrectomy associated with metastatic bone tumor resection is being tested to improve functional status and survival, especially when metastasis involves supporting bones.

## Introduction

Renal cell carcinoma (RCC) represents about 2% of all adult malignant lesions [1]. It is the most common renal neoplasm and accounts for 85% of all solid renal tumors in adults [2]. The clear-cell variety is the most common histologic subtype, accounting for 70% of all RCC [3].

Nearly 20-30% of patients with RCC have distant metastasis at presentation [4], and of these, solitary bone metastasis represents 1.6-3.6% [5]. We report here a case of solitary bone metastasis to the sternum as an initial presentation of clear-cell renal carcinoma in a 56-year-old woman.

## Case presentation

A 56-year-old healthy Caucasian Brazilian woman presented with a six-month history of sternal pain and a two month history of increase in volume of the region, without phlogistic signs, fever, or urinary symptoms.

**Figure 1.**
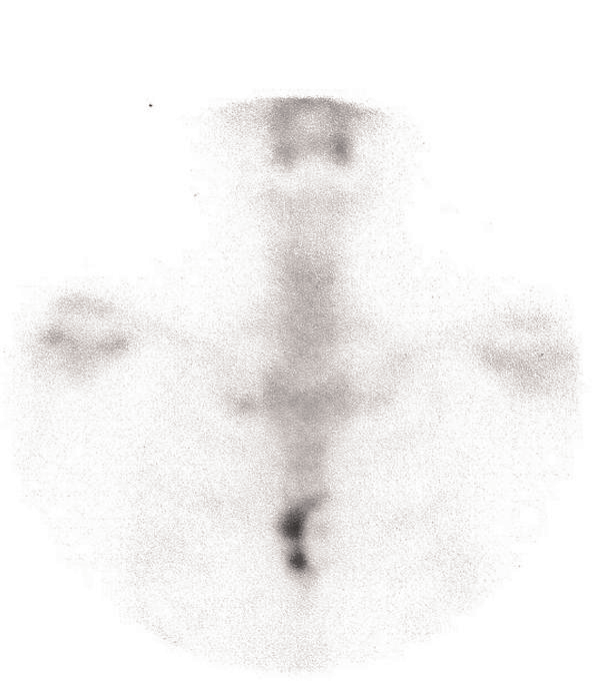
Metastatic bone disease. Bone scintigram shows uptake in the sternal body.

The patient was in good condition and her physical examination was normal, except for a painful palpable mass in the sternal region, without phlogistic signs, occupying almost the entire body of the sternum. Her hemogram, urea, creatinine, liver function tests, and urinalysis were unchanged. Technetium-99 m methylene diphosphonate (MDP) bone scanning showed uptake in the sternal body (Figure 1). She was treated with anti-inflammatory drugs, and instructed to return ten days later, for new assessment. She did not return for the follow-up.

**Figure 2.**
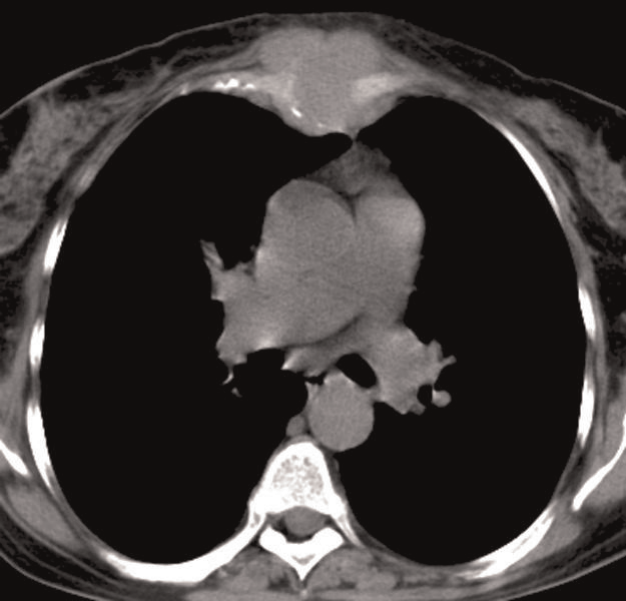
Thoracic CT scan showing soft-tissue mass in the sternum.

**Figure 3.**
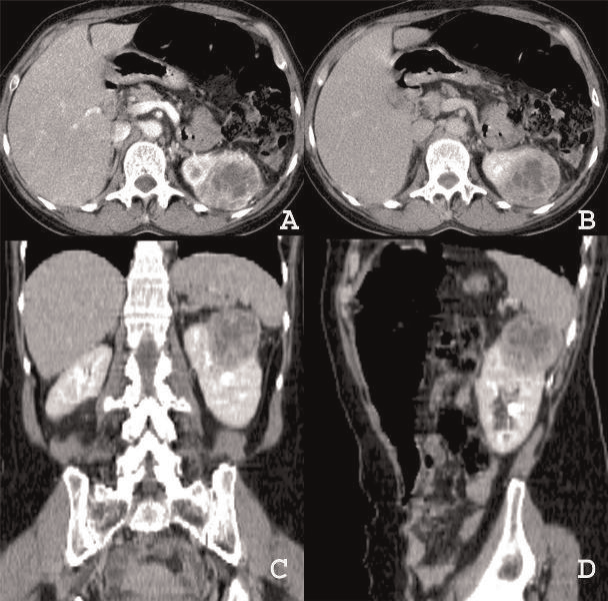
Abdominal CT scan demonstrating a left-sided mass in a 56-year-old woman. The mass was well-defined, exophytic-appearing to arise from the cortex—and heterogeneously and avidly enhancing. **(A)** Corticomedullary and **(B)** Nephrographic phases show typical hypervascularity of the tumor. **(C)** Coronal and **(D)** Sagittal multiplanar reformatation during excretory phase.

Subsequently, the patient noticed increased local volume and worsening pain, and she again sought medical care. Computed tomography (CT) of the chest showed a soft-tissue mass with heterogeneous contrast enhancement, occupying almost all of the sternal body and extending to the subcutaneous tissue, but with no evidence of lung lesions or lymphadenopathy (Figure 2). Because of the possibility that the sternal mass was a metastasis, a search for a primary tumor was undertaken with mammography and abdominal ultrasonography. Sonography showed an exophytic left-sided renal mass measuring about 5 cm in diameter, locally distorting the pyelocaliceal system, without venous thrombus. The result suggested RCC. Likewise, an abdominal CT scan following administration of contrast medium revealed a heterogeneous and avidly enhancing mass in the same location, with hypodense central areas indicative of cystic degeneration or necrosis. The CT scan also revealed local distortion of the pyelocaliceal system, without evidence of venous thrombus or lymphadenopathy (Figure 3).

Preoperative percutaneous biopsy guided by CT scan of both the renal and sternal lesions showed histopathologic diagnosis of clear-cell renal carcinoma. The patient was planned for nephrectomy and radiotherapy of the sternal lesion for palliation of the pain and reduction of the tumor volume. The patient returned for follow-up assessment six months later, complaining of left upper limb paresy/paresthesia. The cervical spine MRI showed lesions compatible with metastasis. She died six months later, with generalized metastasis.

## Discussion

The classic presentation of RCC comprising pain, flank mass, and hematuria occurs in only 10% of cases [6]. Twenty to thirty-nine percent of patients with RCC are asymptomatic, and the diagnosis is made incidentally from a radiologic study obtained for other reasons [6]. Frequently, the first symptoms are from metastatic lesions or paraneoplastic syndromes, including hypercalcemia, erythrocytosis, and non-metastatic hepatocellular enzyme elevation (Stauffer syndrome). Polyneuromyopathy, amyloidosis, anemia, fever, cachexia, weight loss, dermatomyositis, and hypertension also are associated with RCC. In 38-48% of patients with bone metastases, local pain led to the search for and diagnosis of kidney tumors [5].

Accordingly, 25-30% of patients are found to have metastases at diagnosis [7], 25% have locally advanced disease, and 45% have localized disease (stages I and II) [8]. Estimates show that about 60-70% of patients will develop metastases during the course of their illness [9]. The most common sites of metastasis in RCC are the lung (75% of cases), lymph nodes (36%), bones (20%), liver (18%), skin (8%), and brain (8%) [10]. Solitary metastases occur in less than 5% of cases of metastasized disease [7]. Bone infiltration occurs in 50% of patients with multiple organ metastasis, and 15-30% of these lesions are solitary [5].

The tumor size, staging, and histopathologic grading of the nuclei of the tumor are important factors determining the likelihood that metastatic disease develops [11]. Nuclear grading according to the Fuhrman classification system [12] is widely accepted and has been shown to confer prognostic significance. A sharp increase in metastases and a decrease in survival have been noted for patients with lesions that are nuclear grade 3 or 4 [13]. TNM tumor staging is even more predictive than the Fuhrman system: capsular transgression defines a T3 lesion, and T3 or T4 lesions are associated with a significant increase in metastases and a decrease in survival [4].

Nephrectomy remains the standard management for localized disease and now has a role in selected patients with metastatic disease. It may be justified in patients with metastatic disease to improve quality of life or local symptoms and possibly to increase survival [6]. Palliative nephrectomy may be considered for alleviation of pain, hemorrhage, malaise, hypercalcemia, erythrocytosis, or hypertension in patients with metastatic disease [6]. However, it is not justified as a means to induce spontaneous tumor regression as this occurs in less than 1% of cases, most frequently in the lungs [14]. Preoperative embolization can provide symptomatic relief and reduce intraoperative bleeding in large tumors. Treatment of metastasis is usually palliative and aims to provide patient comfort by means of pain relief. Radiation therapy is sometimes used for palliation and is an option to alleviate bone pain associated with metastasis.

Resection of isolated bone metastases together with radical nephrectomy has been advocated when the lesions are larger than 2-3 cm, have destroyed more than 50% of the cortex, or occurs in sustentation bones [9]. Treatment aims to prevent pathological fractures, alleviate pain, increase functional mobility, and prolong survival. Surgical excision of a solitary metastasis may improve survival, especially in cases of lung and bone metastases [4]. Kozlowski [5] showed that two-year survival after surgical excision of solitary synchronous metastatic lesions was 22%.

Immunotherapy of metastatic RCC gave durable remissions in only a minority of patients; the response rate to immunotherapy with interleukin-2 or interferon-α was only 20% [15]. Currently, less than 5% of metastatic RCCs respond to chemotherapy. However, newer agents such as sorafenib and sunitinib have emerged as a promising treatment. These tyrosine kinase inhibitors that specifically inhibit receptors for both vascular endothelial growth factor and platelet-derived growth factor have been shown to improve progression-free survival in metastatic RCC [16].

## Conclusion

Despite technological advances that ought to ensure the early identification of RCC, about 25-30% of patients have metastases at presentation. The prognosis of these patients is poor, especially due to the lack of effectiveness of radiation and chemotherapy. New immunomodulatory agents have been tested, but successes have been infrequent and short-lived. Radical nephrectomy together with resection of metastatic bone tumors and radiation therapy are being tested to improve functional status and survival, especially when metastasis involves supporting bones. These methods can contribute to long-term survival in some patients.

## Abbreviations

CT: computed tomography
RCC: renal cell carcinoma
